# Defining the ferroptotic phenotype of beta cells in type 1 diabetes and its inhibition as a potential antidiabetic strategy

**DOI:** 10.3389/fendo.2023.1227498

**Published:** 2023-08-03

**Authors:** Milica Markelic, Ana Stancic, Tamara Saksida, Ilijana Grigorov, Dragica Micanovic, Ksenija Velickovic, Vesna Martinovic, Nevena Savic, Andjelija Gudelj, Vesna Otasevic

**Affiliations:** ^1^ Department of Cell and Tissue Biology, Faculty of Biology, University of Belgrade, Serbia; ^2^ Department of Molecular Biology, Institute for Biological Research “Siniša Stanković”, National Institute of Republic of Serbia, University of Belgrade, Belgrade, Serbia; ^3^ Department of Immunology, Institute for Biological Research “Siniša Stanković”, National Institute of Republic of Serbia, University of Belgrade, Belgrade, Serbia

**Keywords:** ferroptosis, β-cell death, diabetes, ferroptosis inhibitor, ferrostatin-1

## Abstract

**Introduction:**

Recently, the involvement of ferroptotic cell death in the reduction of β-cell mass in diabetes has been demonstrated. To elucidate the mechanisms of β-cell ferroptosis and potential antidiabetic effects of the ferroptosis inhibitor ferrostatin-1 (Fer-1) in vivo, a mouse model of type 1 diabetes (T1D) was used.

**Methods:**

Animals were divided into three groups: control (vehicle-treated), diabetic (streptozotocin-treated, 40 mg/kg, from days 1-5), and diabetic treated with Fer-1 (1 mg/kg, from days 1-21). On day 22, glycemia and insulinemia were measured and pancreases were isolated for microscopic analyses.

**Results:**

Diabetes disturbed general parameters of β-cell mass (islet size, β-cell abundance and distribution) and health (insulin and PDX-1 expression), increased lipid peroxidation in islet cells, and phagocytic removal of iron-containing material. It also downregulated the main players of the antiferroptotic pathway - Nrf2, GPX4, and xCT. In contrast, Fer-1 ameliorated the signs of deterioration of β-cell/islets, decreased lipid peroxidation, and reduced phagocytic activity, while upregulated expression of Nrf2 (and its nuclear translocation), GPX4, and xCT in β-cell/islets.

**Discussion:**

Overall, our study confirms ferroptosis as an important mode of β-cell death in T1D and suggests antiferroptotic agents as a promising strategy for the prevention and treatment of diabetes

## 1 Introduction

Unlike accidental cell death, which occurs immediately in response to severe chemical, physical, or mechanical insults, regulated cell death (RCD) relies on specific molecular machinery and therefore can be modulated pharmacologically or genetically (1, 2). Rather than focusing on the morphological parameters, the current classification of RCD focuses on the molecular and essential aspects, including signal transduction and the pathophysiological significance of RCD (1, 3). In this context, a necrotic form of RCD named ferroptosis was described more than a decade ago (4). It is manifested by an iron-dependent accumulation of membrane lipid peroxides. Of the processes involved in ferroptosis, the best known is the depletion of the antioxidant peptide glutathione (GSH), which leads to the failure of the lipid peroxide removal capacity of the specific membrane-associated isoform of the GSH-dependent peroxidase, GPX4 (5, 6). The main regulator of intracellular biosynthesis of GSH is a membrane Xc- glutamate/L-cystine antiporter, i.e., its light chain subunit xCT (SLC7a11) (7), which is responsible for the cellular uptake of L-cystine, which is converted to cysteine, a precursor of GSH (8). Cellular susceptibility to ferroptosis is primarily triggered by the imbalance between iron import, storage, and export, which can lead to an increase in the cytosolic labile iron pool (9). Excess cellular iron stimulates ferroptosis either directly by reacting with membrane lipids or via the iron-dependent enzymes involved in reactive oxygen species (ROS) formation and lipid peroxidation, such as lipoxygenases (10, 11). Nuclear Factor Erythroid 2–Related Factor 2 (Nrf2) is considered a master regulator of the expression of the genes related to ferroptosis, including GPX4 and SLC7a11. In addition, Nrf2 has been shown to maintain iron homeostasis by regulating the synthesis and degradation of ferritin (12).

Our understanding of ferroptosis mechanisms and its role in various diseases has been rapidly expanding. This is supported by a plethora of studies suggesting that pharmacological modulation of ferroptosis through its induction (in cancer) or inhibition (in ischemic/degenerative diseases) has potentially significant clinical utility (13). Recently, the involvement of ferroptosis in the etiology and pathogenesis of diabetes and diabetic pathologies has been proposed by several teams, including ours (14–20). Reduction of pancreatic β-cell mass is the most important pathological feature of both type 1 (T1D) and type 2 (T2D) diabetes, and the main cause is considered to be cell death (21). Although the main modules of RCD of β-cells described previously were apoptosis, necrosis, and autophagic RCD (22), in our recent study we confirmed the induction of ferroptosis under diabetogenic conditions *in vitro* (14). In addition, an *in vivo* pilot study presented in the same paper, demonstrated that ferroptosis is an important mode of β-cell death in T1D experimentally induced by streptozotocin (STZ), and suggested the use of a ferroptosis inhibitor ferrostatin-1 (Fer-1) for the protection and survival of β-cells under diabetic conditions. Fer-1 is a free-radical scavenging synthetic antioxidant that inhibits iron-dependent lipid peroxidation (23). Our preliminary *in vivo* results showed that Fer-1 reduced lipid peroxidation, resulting in improved islet size, increased insulin expression, and a decrease in signs of (peri-)insulitis (14). β-Cells are considered to have low antioxidant capacity and are therefore susceptible to oxidative stress (24, 25), justifying the study of the involvement of ferroptosis as a ROS-driven RCD in the pathogenesis of diabetes.

The aim of this work is to elucidate the mechanisms of reduction in β-cell mass under diabetic conditions *in vivo*, including characterization of the ferroptotic phenotype in situ. In addition, we aimed to define the benefits of ferroptosis inhibition in the endocrine pancreas under diabetic conditions. For this purpose, the same ferroptosis inhibitor as in our previous *in vitro* and *in vivo* pilot study, Fer-1, was selected and. a thorough microscopic examination of pancreatic tissue from diabetic mice was performed.

## 2 Materials and methods

### 2.1 Experimental design

For this study, 8–10-week-old male C57BL/6 mice were housed in the animal facility of the Institute of Biological Research “Sinisa Stankovic” (IBISS) with unlimited access to standard food and tap water. All experimental procedures were approved by the IBISS Ethics Committee (App. No. 323-37-11487/2021-05) in accordance with Directive 2010/63/EU. Animals were divided into three groups (n = 8): diabetic (DM), diabetic Fer-1-treated (DM + Fer-1), and untreated control animals (Ctrl). For induction of diabetes, multiple low doses of STZ (40 mg/kg body mass; S0130, Sigma-Aldrich, St. Louis, MI, USA) were injected intraperitoneally (i.p.) for 5 consecutive days (days 1-5) as previously described (14). Fer-1 (1 mg/kg body mass, SML 0583, Sigma-Aldrich), dissolved in dimethyl sulfoxide (DMSO, D8418, Sigma-Aldrich) and diluted with phosphate-buffered saline (PBS), was administered i.p., starting from day 1 to 21. To avoid possible interference, the injections of STZ and Fer-1 were administered 3 h apart. The control group received the diluents in the same amount. Twenty-four hours after the last STZ and Fer-1 administration, the animals fasted for 4 h were euthanized by cervical dislocation between 9:00 and 9:30 AM. Blood and pancreas samples were collected and routinely processed for biochemical or microscopic analyses.

### 2.2 Measurement of serum glucose and insulin levels

Immediately after blood collection, serum was processed (26) and stored at -80°C until analysis of serum glucose level (Biochemical Laboratory “Beograd”, Belgrade, Serbia) and serum insulin level (by radioimmunoassay, INEP, Belgrade, Serbia).

### 2.3 Microscopic examination

Immediately after dissection, pancreas pieces were sectioned and fixed overnight at 4°C in 10% formaldehyde, routinely processed for embedding in paraffin blocks, and cut into 5 μm thin sections for microscopic analyses.

#### 2.3.1 Histological, morphometric, and stereological analyses

For routine histological analysis and for morphometric analysis of islet surface profiles, tissue sections were routinely stained with hematoxylin and eosin (HE). In addition, AZAN trichrome staining was performed to determine the presence/stage of islet fibrosis. The islet fibrosis, reflecting extensive collagen deposition surrounding/infiltrating islets, is visible as intensive blue staining (27) and was scored as: 0 – absent, 1 – mild, 2 – moderate, 3 – massive. For morphometric measurement of the islet surface area, approximately 50 islets per group were analysed using Image J software (NIH, USA) at a lens magnification of x 40. To detect the accumulation of iron (Fe^3+^), Pearl’s Prussian Blue staining was performed in a 2% potassium ferrocyanide solution followed by Nuclear Fast Red counterstaining. A positive reaction was detected as blue intracellular accumulation. For detection of lipofuscin, sections were stained with a 0.35% Sudan Black B (SBB) solution in 70% ethanol followed by Nuclear Fast Red counterstaining. A positive reaction was detected as brown to black intracellular granular accumulations. All sections were mounted in DPX mounting medium (Sigma-Aldrich) and examined using a DMLB microscope (Leica Microsystems, Wetzlar, Germany).

#### 2.3.2 Immunohistochemistry and immunofluorescence

To determine the expression and localization of insulin, glucagon, pancreas/duodenum homeobox protein 1 (PDX-1), 4-hydroxynonenal (4-HNE), Nrf2 and its downstream targets: GPX4, xCT, heme oxygenase-1 (HO-1) and peroxiredoxin-2 (PRDX-2) in the endocrine pancreas, immunohistochemical or immunofluorescence staining was performed. To analyze the localization of α- and β-cells, a comparative analysis of serial tissue sections immunostained against insulin and glucagon (i.e., “mirror**”** technique) was performed. Five μm thick pancreatic sections were deparaffinized with xylene and rehydrated in graded ethanol. Antigen retrieval in citrate buffer (pH 6.6, microwave boiling, 5 min), blocking of endogenous peroxidase activity (3% H_2_O_2_ in methanol, 10 min), and blocking of nonspecific binding with bovine serum albumin (5% BSA in PBS, 30 min) were performed before incubation of primary antibodies. Samples were incubated overnight at 4°C with the following primary antibodies diluted in 1% BSA in PBS: rabbit anti-insulin (1:100, sc-9168, Santa Cruz Biotechnology, TX, USA), anti-glucagon (1:300, sc-13091, Santa Cruz Biotechnology), anti PDX-1 (1:1000; sc-25403, Santa Cruz Biotechnology), anti-4-HNE (ab46545, 1:500; Abcam, Cambridge, UK), anti-Nrf2 (ab31163, 1:100, Abcam), anti-GPX4 (ab125066, 1:100, Abcam), anti-HO-1 (ab13243, 1.25:1000, Abcam) and mouse anti-PRDX-2 (sc-515428, 1:200, Santa Cruz Biotechnology). After rinsing with PBS, the sections were incubated with secondary goat anti-rabbit or anti-mouse antibody, depending on the host of the primary antibody (ab97051 and ab6789, 1:1000, Abcam, UK) for one hour at room temperature. The final reaction product was visualized after incubation in DAB chromogen solution (K 3408, Dako liquid DAB+ Substrate Chromogen Substrate System, Agilent Technologies, Santa Clara, CA, USA). After counterstaining with hematoxylin, slides were mounted in DPX and examined with a light microscope (Leica Microsystems). For the quantification of immunopositivity of islets to Nrf2, GPX4, 4-HNE, HO-1, PDX-1 and PRDX-2, Color deconvolution tool in Image J (NIH) was applied (H-DAB setup) and DAB (brown signal) images were used to determine mean grayscale value of islets per group. Arbitrary values were calculated as 1000/greyscale level to obtain a direct proportionality between the signal intensity and the values obtained. β- and α-cell ratio was calculated by counting insulin- and glucagon-positive cells in islets. The values were presented as number of cells per islet area (1000 μm^2^).

To detect the localization of xCT in pancreatic β-cells, double immunofluorescence labelling of xCT and insulin was performed. After routine deparaffinization, rehydration, antigen retrieval with citrate buffer, and protein blocking (5% normal goat serum in 1% BSA in PBS-Tween), the mixture of goat anti-xCT (1:40, sc79360, Santa Cruz Biotechnology) and rabbit anti-insulin antibodies (1:100, sc9168, Santa Cruz Biotechnology) was applied and incubated overnight at 4°C. After thorough washing, the sections were incubated with the appropriate secondary antibodies: anti-goat Alexa Fluor 488 (1:200; A-11055, Thermo Fisher Scientific, Waltham, MA, USA) and anti-rabbit Alexa Fluor 647 (1:200; ab 150079, Abcam). Antibodies were diluted in 1% BSA in PBS-Tween and incubated for 30 min at room temperature. After washing, sections were embedded in Mowiol (Sigma-Aldrich) and analysed on the SP5 confocal microscope (Leica Microsystems). Average xCT immunopositivity of β-cells was measured in LAS AF software (Leica Microsystems).

#### 2.3.3 TUNEL staining

To determine whether cell death-related DNA fragmentation occurs *in situ* under diabetic conditions, fluorescent TUNEL staining (*In Situ* Cell Death Detection Kit, Fluorescein; Roche Applied-Science) was performed according to the manufacturer’s protocol. After deparaffinization and rehydration, slides were incubated with proteinase K solution for 30 min at 37°C, washed in PBS for 2 min, and incubated with TUNEL reaction mixture for 1 h at 37°C after blocking. After washing, slides were counterstained with propidium iodide, washed, and mounted with Mowiol mounting medium (Sigma-Aldrich) for analysis on the SP5 confocal microscope (Leica Microsystems). Average nuclear TUNEL fluorescence intensity of islet cells was measured in LAS AF software (Leica Microsystems).

### 2.4 Statistical analysis

Statistical analysis was performed using GraphPad Prism software (GraphPad Software, San Diego, CA, USA). To test the data for normality, the Kolmogorov-Smirnov test was used. If the F test indicated an overall difference, one-way analysis of variance (one-way ANOVA) was performed, followed by Tukey’s multiple comparison test. Statistical significance was set at p < 0.05.

## 3 Results

**Figure 1:**
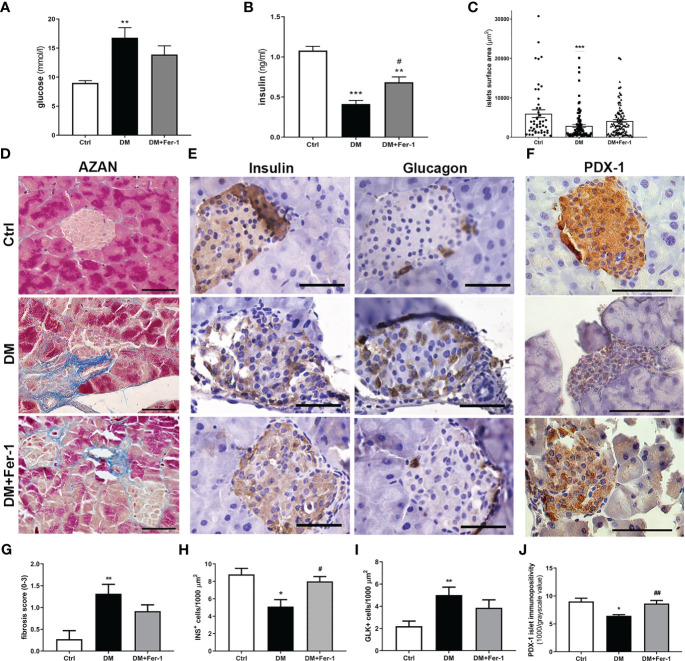
**(A)** Serum glucose levels and **(B)** serum insulin levels at day 22 of experiment; **(C)** islets surface area; **(D)** representative micrographs of islets, AZAN trichrome staining; **(E)** comparative immunohistochemical detection of insulin- and glucagon-positive cells at serial pancreatic sections; **(F)** immunohistochemical detection of PDX-1; **(G)** fibrosis score; **(H)** ratio of insulin-positive β (INS^+^) and **(I)** glucagon-positive α cells (GLK^+^); **(J)** average PDX-1 immunopositivity in islets of Langerhans from control (Ctrl), diabetic (DM), and Fer-1-treated diabetic animals (DM+Fer-1). Magnification and scale bar **(D–F)**: ×40, 50 μm. Statistical significance **(A–C, G–J)**: *p < 0.05, **p < 0.01, ***p < 0.001 – in comparison to Ctrl; ^#^p < 0.05, ^##^p < 0.01 – in comparison to DM.

As expected, at the end of the experiment (day 22), the mean serum glucose and insulin levels were significantly altered in the diabetic animals (**Figures 1A, B**). Concurrent treatment with Fer-1 slightly lowered the blood glucose level toward the control value, and significantly increased insulin level above the value measured in the diabetic group. Consistent with the serum glucose and insulin levels, a decrease in the mean islet size was less pronounced in the Fer-1-treated diabetic animals than in the untreated diabetic animals (**Figure 1C**). Additionally, fibrosis observed around and inside islets of diabetic mice also tended to decline toward control level of collagen depositions when treatment with Fer-1 was applied (**Figures 1D, G**),

Comparative immunohistochemical detection of insulin- and glucagon-positive cells in serial sections of pancreatic tissue showed fewer insulin-positive β-cells in the islets of diabetic mice than in the islets of control animals (**Figures 1E, H**). In addition, the proportion of glucagon-positive α-cells increased (**Figure 1I**), followed by their migration from the periphery of the islet to its center. Fer-1 treatment of diabetic mice significantly increased the proportion of insulin-positive cells. Moreover, the α-cells are slightly less numerous and more likely to be located in the periphery of the islets in this group.

Immunohistochemical detection of PDX-1 in islets of Langerhans showed a sharp decrease in its expression in diabetic mice (**Figures 1F, J**). In diabetic animals treated concomitantly with Fer-1, PDX-1 immunoexpression of islets increased, showing strong immunopositivity of the majority of islet cells, similar to that in controls. In addition, mild immunopositivity of exocrine acinar cells was detectable only in this group.

**Figure 2:**
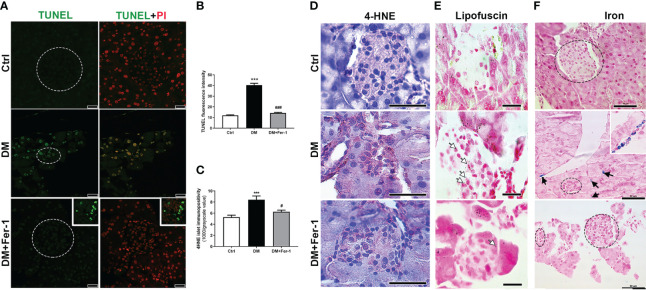
Detection of **(A)** DNA fragmentation (green, TUNEL staining) combined with PI staining of nuclei (red) in islets of Langerhans (encircled), insert – a detail of a lymph node adjacent to pancreas; **(B)** quantification of DNA fragmentation inside islet nuclei (TUNEL fluorescence); **(C, D)** immunohistochemical detection and quantification of 4-HNE in pancreatic islets from control (Ctrl), diabetic (DM), and Fer-1-treated diabetic animals (DM +Fer-1); **(E)** Sudan III staining – lipofuscin detection inside islet cells (white arrows); **(F)** Pearl’s staining – phagocytic iron (Fe^3+^)-loaded cells in exocrine pancreas of diabetic animals (black arrows and insert) –iron-loaded cells;. Magnification and scale bar: **(A)** ×63, 25 μm; **(D, F)** ×40, 50 μm; **(E)** ×100, 20 μm.

To detect signs of DNA fragmentation that might indicate the presence of dying cells, TUNEL staining was performed (**Figure 2A**). Although no signs of pyknosis or apoptotic bodies were detected in the islets of all groups, strong staining of the islet nuclei is evident in the DM group, indicating increased DNA fragmentation. Treatment with Fer-1 reduced this DNA damage, as evidenced by a lower TUNEL signal in the islet nuclei (**Figures 2B**). In addition, immunohistochemically, more 4-HNE adducts were noted in the endocrine pancreas of diabetic animals, indicating the increased lipid peroxidation (**Figure 2D**) as confirmed by the appearance of more islet cells with lipofuscin granules (**Figure 2E**). In these animals pancreatic 4-HNE immunopositivity was particularly pronounced in the islets of Langerhans and blood vessels. Increased 4-HNE immunopositivity of islets was decreased by Fer-1 treatment, thusconfirming lower lipid peroxidation in the pancreas of these animals (**Figure 2C**). In addition, numerous phagocyte-like cells with iron-containing phagosomes/lysosomes were detectable in the pancreas of the diabetic animals (**Figure 2F**). Their presence was rarely detectable in Fer-1-treated diabetic animals. This indicates increased phagocytic activity in the pancreas of diabetic animals, which probably serves to ingest and remove dead islet cells.

**Figure 3:**
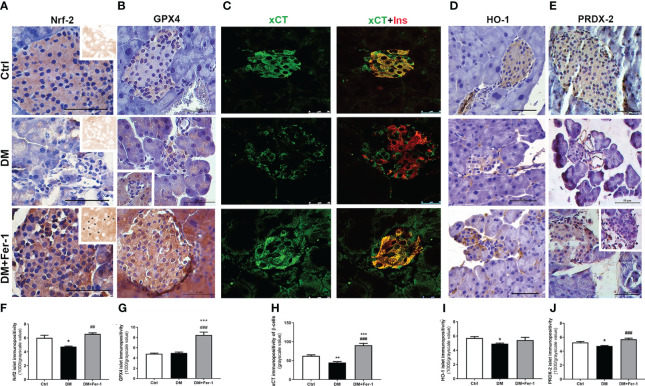
Immunohistochemical detection and quantification of islet immunopositivity to **(A, F)** Nrf2 (insets on **(A)** – DAB channel of the original images, asterisk showing Nrf2 nuclear immunopositivity) **(B, G)** GPX4, **(D, I)** HO-1 and **(E, J)** PRDX-2. Immunofluorescence colocalization of **(C)** xCT (green) and insulin (red) in islets of Langerhans from control (Ctrl), diabetic (DM), and Fer-1-treated diabetic animals (DM+Fer-1) and **(H)** xCT immunopositivity of β-cells. Magnification and scale bar: **(A, B, D, E)** ×40, 50 μm; **(C)** ×63, 25 μm. Statistical significance **(F–J)**: *p < 0.05, **p < 0.01, ***p < 0.001 – in comparison to Ctrl; ^##^p < 0.01, ^###^p < 0.001 – in comparison to DM.

Nrf2 and GPX4 immunopositivity of islet cells is shown in **Figures 3A and B**, respectively. A homogeneous distribution of Nrf2 immunopositivity is detectable in the islet cells of the control animals, with few Nrf2-positive nuclei. In the DM group, Nrf2 immunopositivity of islet cells decreased, whereas Fer-1 treatment of diabetic animals resulted in a sharp increase in Nrf2 immunopositivity that exceeded control level (**Figure 3F**), including more Nrf2-positive nuclei (**Figure 3A** **inset**). GPX4 immunopositivity of control islets is weak, although rim cells (which correspond to α-cells based on their localization) are somewhat more GPX4-positive. A similar pattern is seen in the diabetic group, with slightly increased immunopositivity of surrounding acinar cells (**Figure 3B** **inset**) in contrast to the DM+Fer-1 group, in which strong immunopositivity of all pancreatic endocrine tissue was detected. The differences in the islet immunopositivity to GPX4 among the groups was confirmed by the quantitative analysis (**Figure 3G**).

To analyze the xCT immunopositivity of the islet cells and to determine whether the experimental treatments used altered the expression of xCT in the β-cells, co-staining of xCT and insulin was performed (**Figure 3C**). As shown, islet cells from the control animals are strongly positive for xCT. In the islet cells of the diabetic animals, xCT immunopositivity decreased, with the greatest decrease in β-cells, whereas Fer-1 treatment significantly increased immunoexpression in these cells, even above the control level (**Figure 3H**).

In addition to GPX4 and xCT, immunohistochemical detection of HO-1 and PRDX-2, the additional Nrf2 downstream targets, was performed (**Figures 3D, E**). The results showed a similar pattern for both antioxidant enzymes in the pancreatic islets, with decreased immunopositivity in the DM group, which reverted to control levels in most of the islets when DM animals were treated simultaneously with Fer-1 (**Figures 3I, J**)

## 4 Discussion

**Figure 4:**
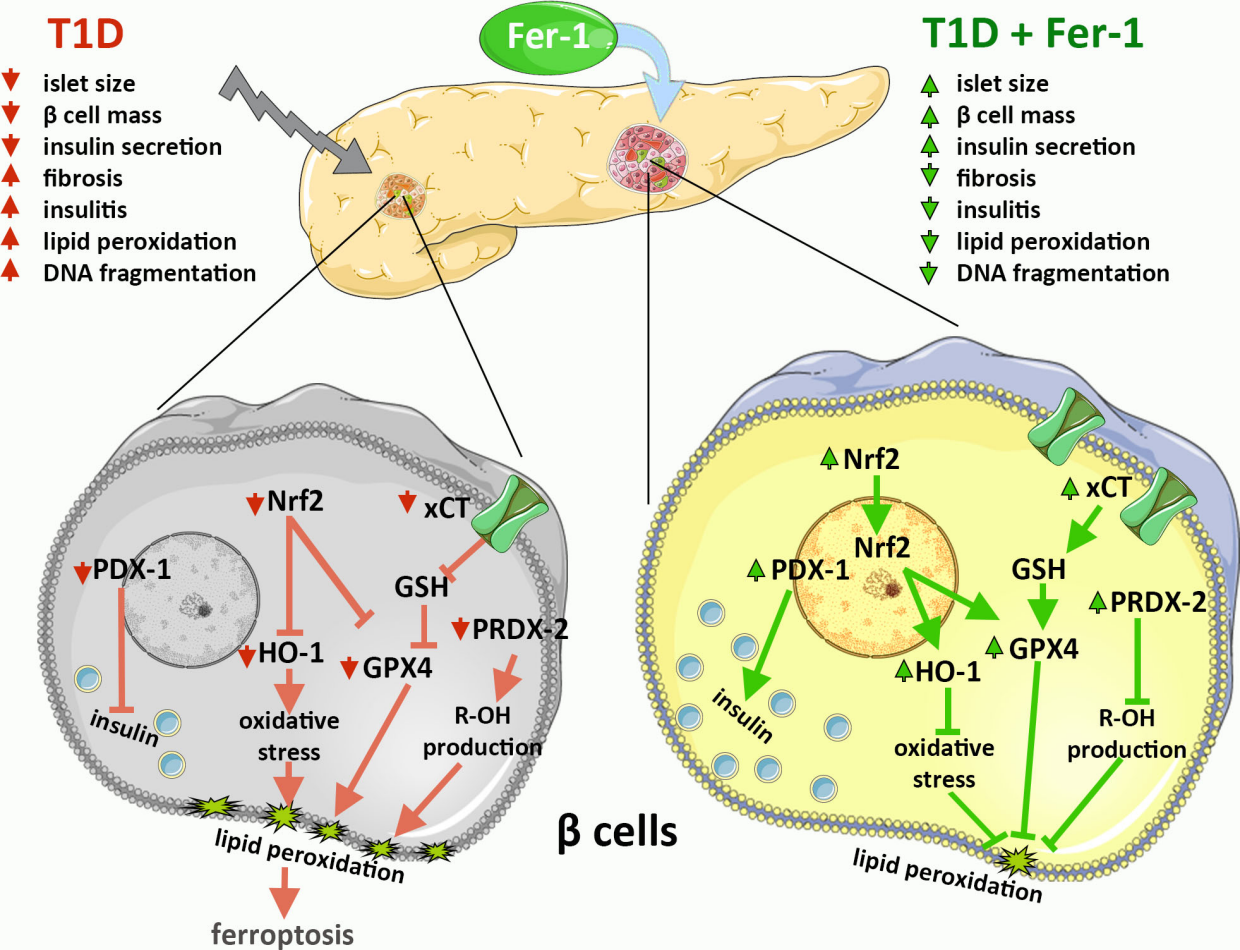
Graphical summary of the results on the contribution of β-cell ferroptosis to the development and pathogenesis of T1D via modulation of Nrf2 and its downstream targets and its inhibition by ferrostatin-1 (Fer-1).

Previous evidence, including ours, suggests the involvement of ferroptosis in the development and progression of diabetes and diabetic complications (14–20). However, data on possible protective effects of antiferroptotic agents in β-cells *in vivo* are still unclear. To elucidate the mechanisms of islet of Langerhans deterioration in T1D and the benefits of using Fer-1 as an antiferroptotic agent in the treatment of this disease, a thorough microscopic examination of pancreatic tissue from diabetic mice was performed. The results of the present study are summarized graphically in **Figure 4**.

The analyses of serum levels of insulin and glucose showed the beneficial effects of Fer-1 in diabetic animals, as levels of both parameters changed toward control values. This is consistent with our previous results (14), in which it was shown that glycemia in diabetic animals treated with Fer-1 tended to recover to physiological levels. At the histological level, the reduced hyperglycemia is associated with a slight improvement in islet size and reduced infiltration of immune cells (insulitis/periinsulitis), as we also showed in our recent work (14). This is followed by a partial restoration of the abundance of insulin-positive β-cells, followed by increased insulin immunopositivity of individual cells when compared to the control, presumably to compensate for the reduction in the number of β-cells.

Moreover, the diabetes-induced reduction of β-cells in islets of Langerhans is followed by the increased appearance of glucagon-positive α-cells migrating to the center of the islet. While this confirms the efficacy of the T1D model used, in which β-cells are selectively destroyed, it also argues for α-cell preservation or even hyperplasia, probably as a loss of insulin-driven paracrine action on neighboring α-cells (28, 29). The beneficial effects of Fer-1 treatment in T1D demonstrated in this study include normalization of the ratio and distribution of α- and β-cells in islets.

One of the parameters presented here for the first time, which speaks in favor of the beneficial effects of Fer-1 on β-cell survival and health is the restoration of PDX-1 protein immunoexpression to the control level. PDX-1 is considered as one of the major transcription factors involved in pancreatic development and maintenance of mature β-cell survival and function, including insulin synthesis and secretion (30–33). Its downregulation in β-cells in diabetes, has been shown to be involved in the dysfunction and death of β-cells (34–36).

After we have shown the improval of general parameters of β-cell mass and health in diabetic animals co-treated with Fer-1, we aimed to quest for the signs of cell death and proferroptotic damage in islets of diabetic animals as well as to investigate their eventual improval after the Fer-1 treatment. Despite a severe reduction in islet size and β-cell mass in the pancreas of diabetic mice, neither apoptotic nor necrotic cells were detected in islets of Langerhans by TUNEL staining. However, mild DNA fragmentation of islet cells was detected. This phenomenon is already described in diabetic β-cells and is implicated in the development and the exacerbation of both T1D and T2D (37, 38). The lack of morphologic evidence of dead islet cells could be explained by their rapid removal by phagocytes from the population of infiltrating immune cells (39, 40). Consistent with this suggestion, numerous macrophage-like cells loaded with iron depositions are found in the pancreas of diabetic mice. Fer-1 treatment however, improved both of these parameters, decreasing the signs of DNA damage of islet cells and presence of iron-loaded cells in the pancreatic tissue.

Further we have confirmed the increased accumulation of lipid peroxides in β-cells of diabetic animals, as demonstrated by increased lipofuscin accumulation and 4-HNE immunopositivity in pancreatic islet cells. The detected signs of islet cell damage, all of which were reduced by Fer-1, suggest the role of ferroptotic cell death in the elimination of β-cells in T1D, as previously shown in our study (14).

To confirm this assumption, an analysis of the immunoexpression and localization of the major contributors to the antiferroptotic pathway in the cell – Nrf2, GPX4, and xCT - was performed. From the literature, Nrf2 responds differently during the onset and progression of diabetes. Namely, in the early stages, Nrf2 is upregulated to respond to increased oxidative stress and overcome damage (41). In chronic hyperglycemia, sustained oxidative stress leads to inhibition of Nrf2 nuclear translocation, stimulation of Nrf2 degradation, and impaired activation of antioxidant genes, contributing to ROS accumulation (42–45). As shown here, on day 22 of the experiment immunoexpression of Nrf2 was reduced or even undetectable microscopically in islet cells of diabetic animals. Because Nrf2 is an antioxidant transcription factor involved in the metabolism of GSH (46–48), lipids (49) and iron (50, 51), its inactivation and/or suppression are considered important steps in the development and outcomes of ferroptosis (52). Consistent with decreased Nrf2 immunoexpression in islet cells in diabetes, a similar pattern has been demonstrated for GPX4, a membrane-associated member of the GPX family (50) that is one of the major downstream target enzymes of Nrf2 activation. GPX4 immunoexpression was greatly reduced in centrally located islet cells (β-), whereas it was unchanged in marginal cells (α-). GPX4 is considered to be the major enzyme involved in the removal of ROS-driven membrane lipid peroxides in the context of ferroptotic cell death (5, 53, 54), as shown by others and our team (14, 15, 55–57). This enzyme has previously been shown to be highly expressed and important for the survival of β-cells (58), suggesting the importance of protecting these cells from oxidative degradation of their membrane lipids.

Along with the decrease of GPX4 protein expression in islets of diabetic animals, it seems evident that its lipid peroxide-removing capacity is impaired due to decreased GSH bioavailability as previously confirmed (59). Namely, there was also a decline in the protein expression of xCT in β-cells of diabetic mice, as evidenced by our immunofluorescence results. L-cystine import by xCT is considered the rate-limiting step in the GSH biosynthesis and GPX4 activity (5, 60).

To further investigate the effects of Nrf2 downregulation in islet cells, we additionally analysed the immunoexpression of HO-1 and PRDX-2 (61), Nrf2 downstream targets involved in antioxidant protection of β-cells and their survival. HO-1 plays an important role against iron-overload-induced stress (62–64), whereas PRDX-2 is a selenium-independent glutathione peroxidase that eliminates peroxides (65, 66). In the case of both enzymes, DM decreased their islet immunoexpression, which was restored by Fer-1.

Altogether, decrease in Nrf2, along with its downstream targets – GPX4, xCT and HO-1, followed by the accumulation of lipid peroxides (as discussed above) in islet cells, suggest ferroptosis as an important mode of β-cells removal in T1D. To our knowledge, the participation of PRDX-2 downregulation in ferroptosis of islet cells is demonstrated for the first time in diabetes, although PRDX family is already shown to protect different cells from ferroptosis (67).

Regarding the effects of Fer-1 on these main cellular contributors involved in the enzymatic degradation of membrane lipid peroxides in the islets of Langerhans of diabetic animals, we have demonstrated upregulated expression and increased nuclear translocation of Nrf2, thus demonstrating activation of the Nrf2 pathway by Fer-1. This was followed by the upregulated expression of GPX4, xCT, HO-1 and PRDX-2 in β-cells. To date, Fer-1 is known to be one of the most potent inhibitors of ferroptosis, exhibiting high potency as a free radical scavenging antioxidant, with particularly high potency in phospholipid bilayer membranes compared with other antioxidants (68), and the mechanism by which it activates Nrf2 is currently unknown but certainly worthy of further investigation. All of the mentioned improvements in Nrf2 signaling pathway argue for the observed antiferroptotic activity of Fer-1 in β-cells *in vivo*, which could be considered as a potential antidiabetic strategy to preserve their functionality and mass. The protective effects of Fer-1 in both diabetic endocrine pancreas and liver (14, 15), give us insight into its actions and allow the selection of additional feropttosis inhibitors in future studies, such as liproxtatin-1 which has been shown to be highly suitable for *in vivo* application (69). Overall, our *in vivo* microscopic study confirms the important contribution of ferroptosis of β-cells in the development and pathogenesis of T1D via modulation of Nrf2 and its downstream targets. Moreover, our results suggest antiferroptotic agents as a promising therapeutic strategy for the prevention and treatment of diabetes.

## Data availability statement

The raw data supporting the conclusions of this article will be made available by the authors, without undue reservation.

## Ethics statement

The animal study was reviewed and approved by Ethics Committee of the Institute of Biological Research “Sinisa Stankovic”, National Institute of Republic of Serbia, University of Belgrade, Belgrade, Serbia.

## Author contributions

MM: methodology, validation, formal analysis, investigation, resources, writing - original draft preparation, review and editing, visualization. AS: conceptualization, methodology, validation, formal analysis, investigation, writing - review and editing, visualization. TS: methodology, resources, writing - review and editing. IG: conceptualization, formal analysis, resources, writing - review and editing. DM: methodology, investigation. KV: validation, resources, writing - review and editing. VM: validation, formal analysis. NS: methodology, investigation. AG: investigation, formal analysis. VO: conceptualization, methodology, validation, investigation, resources, data curation, supervision, project administration, funding acquisition. All authors contributed to the article and approved the submitted version.
